# Supplementary material for: A higher order PUF complex is central to regulation of C. elegans germline stem cells
Source: Nat Commun. 2025 Jan 2;16:123. doi: 10.1038/s41467-024-55526-x (PMC11696143; doi:10.1038/s41467-024-55526-x)
Supplement: Supplementary file 1 — Supplementary Information [file 41467_2024_55526_MOESM1_ESM.pdf]

Supplementary Information:

A higher order PUF complex is central to regulation of *C. elegans* germline stem cells

Qiu, et al.

Supplementary Information

Supplementary Table 1. Crystallographic data collection and refinement statistics.

	**FBF-2 RBD/FBEa***
**Data collection**	
Space group	$P6_1$
Unit cell dimensions<br>a, b, c (Å)<br>α, β, γ (°)	 99.0, 99.0, 107.2<br>90, 90, 120
Resolution range (Å)[1]	50-2.20<br>(2.24-2.20)[2]
Unique reflections3	29825 (1511)
Multiplicity	5.0 (4.7)
Completeness (%)	99.4 (99.8)
Mean I/sigma(I)	10.6 (1.0)
Wilson B-factor	51.8
R-meas	0.16 (1.24)
R-pim	0.07 (0.56)
CC1/2	0.99 (0.50)
**Refinement**	
Resolution range (Å)	36.27-2.20<br>(2.25-2.20)[2]
Reflections used in refinement	29170
Reflections used for R-free	2185
R-work	0.197 (0.342)
R-free	0.232 (0.371)
Number of atoms	
protein	3201
RNA	212
solvent	92
Average B-factors ($Å^2$)	
protein	68.3
RNA	80.2
solvent	60.3
RMSD bonds (Å)	0.002
RMSD angles (°)	0.405

[1]One crystal was used for data collection.

[2]The highest-resolution shell is shown in parentheses.

[3]Statistics for the highest-resolution shell are shown in parentheses.

Supplementary Table 2. Statistical significance for GLD-1 quantitation comparisons

Figure	Strains compared	Region (µm from distal end)	p-value	Sig[1]
	**Endogenous**			
3e	FBEa*^{m} (n=52)<br>vs control (n=40)	0-10<br>70-80<br>90-100	<0.001<br>0.644<br>0.751	***<br>ns<br>ns
3f[2]	FBEa^{m} (n=64)<br>vs control (n=37)	0-10<br>70-80<br>90-100	<0.001<br>0.365<br>0.416	***<br>ns<br>ns
3f, h	FBEa^{m} (n=64)<br>vs FBEa^{m} FBEa*^{m} (n=26)	0-10<br>70-80<br>90-100	0.663<br>0.001<br><0.001	ns<br>**<br>***
3h	FBEa^{m} FBEa*^{m} (n=26)<br>vs control (n=20)	0-10<br>70-80<br>90-100	<0.001<br>0.036<br>0.006	***<br>*<br>**
3i	FBEa*^{m} FBEb^{m} (n=49)<br>vs control (n=37)	0-10<br>70-80<br>90-100	0.018<br>0.025<br>0.009	*<br>*<br>**
3e, h[3]	FBEa*^{m} FBEb^{m}(n=49)<br>vs FBEa*^{m} (n=31)[3]	0-10<br>70-80<br>90-100	0.568<br>0.007<br>0.002	ns<br>**<br>**
	**Reporter**			
S3b[2]	FBEa^{m} (n=24) vs wt (n=25)	0-10<br>70-80<br>90-100	<0.001<br>0.693<br>0.350	***<br>ns<br>ns
S3b	FBEa^{m} FBEa*^{m} (n=19)<br>vs wt (n=25)	0-10<br>70-80<br>90-100	<0.001<br>0.010<br>0.003	***<br>*<br>**

[1] Significance: *** < 0.001; ** < 0.01; * < 0.05; ns > 0.05; p-values using a two-sided unpaired t-test assuming equal variance.
[2] Endogenous FBEa data from Carrick et al, 2024. Reporter FBEa data from this work.
[3] Separate data set from Fig. 3d, done in same experiment as FBEa*^{m} FBEb^{m}

Supplementary Table 3. Cryo-EM data collection statistics.

	1 FBF-2	2 FBF-2
**Data collection and processing**		
EMDB code	EMD-45096	EMD-45097
Magnification	45,000	
Voltage (kV)	200	
Electron exposure ($e^-/Å^2$)	54	
Defocus range (μm)	-1.0 to -2.5	
Pixel size (Å)	0.932	
Symmetry imposed	C1	
Initial particle images (no.)	1,263,785	
Final particle images (no.)	252,126	110,843
Map resolution (Å)<br>FSC threshold	4.4<br>0.143	6.4<br>0.143
Map resolution range (Å)	4.1-20.8	5.8-14.6

Supplementary Table 4. RNA sequences used in the EMSA RNA-binding experiments.

RNA	Sequence (with 3´-Cy5)[1]
FBEa-FBEa*	AUCA**UGU**GCCAUACAUCA**UGUU**GCCAUUU
FBEa^{m}-FBEa*	AUCAACAGCCAUACAUCA**UGUU**GCCAUUU
FBEa-FBEa*^{m}	AUCA**UGU**GCCAUACAUCAACA**U**GCCAUUU
FBEa^{m}-FBEa*^{m}	AUCAACAGCCAUACAUCAACA**U**GCCAUUU
FBEa*	AUCA**UGUU**GCCAUUU

[1]Mutations in red. FBE sequences underlined.

Supplementary Table 5. Sequences of guide RNAs and repair templates to create CRISPR alleles.

Description	Guide (5´-3´)	Repair template (5´-3´)
FBEa	aaaaatggcaacatgatgta	gttcgttctcaccattttaggttaccatagaatcaACAg<br>cGatacatcatgttgccattttttccccctctcatctcccc
FBEa*	aaaaatggcaacatgatgta	gttcgttctcaccattttaggttaccatagaatcatgtgcc<br>atacatcaACAtgccattttttccccctctcatctcccc
FBEa-FBEa*	aaaaatggcaacatgatgta	gttcgttctcaccattttaggttaccatagaatcaACAg<br>cGatacatcaACAtgccattttttccccctctcatctcc<br>cc
FBEb	ataacTGTgaaaaataaagg	cccattcatactacctcgaatgccaaagcacccccttttatttt<br>cACAgttatcttaacgctaacccctgtagaatcttcccggt

Supplementary Table 6. Strains used in this manuscript.

	**Strain name**	**allele**	**Comments**
N2			
sur-5	JK4864	*qIS147*	sur-5::GFP marked wt control
unc-119(ed3) III; teIs1 IV	TX189	*teIs1*	oma-1::GFP prevents GFP silencing
**Endogenous *gld-1* Crispr alleles**			**Oligos to detect FBE mutations (5´-3´)**
gld-1 FBEa^{m} [1]	JK6540	*q1242*	slc299 GAAGTACCCAACAACCACTTCG<br>prHJS401 TGGCAACATGATGTATCGCTGT
gld-1 FBEa*^{m}	JK6531	*q1234*	slc299 GAAGTACCCAACAACCACTTCG<br>slc301 GAGAGGGGGAAAAAATGGCATGT
gld-1 FBEb^{m} [1]	JK6568	*q1257*	slc299 GAAGTACCCAACAACCACTTCG<br>slc302 GGGTTAGCGTTAAGATAACTGT
gld-1 FBEa^{m}-FBEa*^{m}	JK6541	*q1243*	Use primer sets for FBEa and a*
gld-1 FBEa*^{m}-FBEb^{m}	JK6736	*q1297*	Use primer set for FBEa* and b
gld-1 FBEa^{m}-FBEb^{m} [1]	JK6602	*q1271*	Use primer sets for FBEa and b
**Reporter strains and Crispr alleles**			
rajSi50 FBEwt [2]	JK6694	*rajSi50*	slc314 GTCACCAAGTACACTTCCAGCAAG<br>slc301 GAGAGGGGGAAAAAATGGCATGT
rajSi50 FBEa^{m} [1]	JK6551	*q1274*	slc314 GTCACCAAGTACACTTCCAGCAAG<br>prHJS401 TGGCAACATGATGTATCGCTGT
rajSi50 FBEa^{m}-FBEa*^{m}	JK6639	*q1275*	Use primer sets for FBEa and a*

[1]Carrick et al. [2] Theil et al.

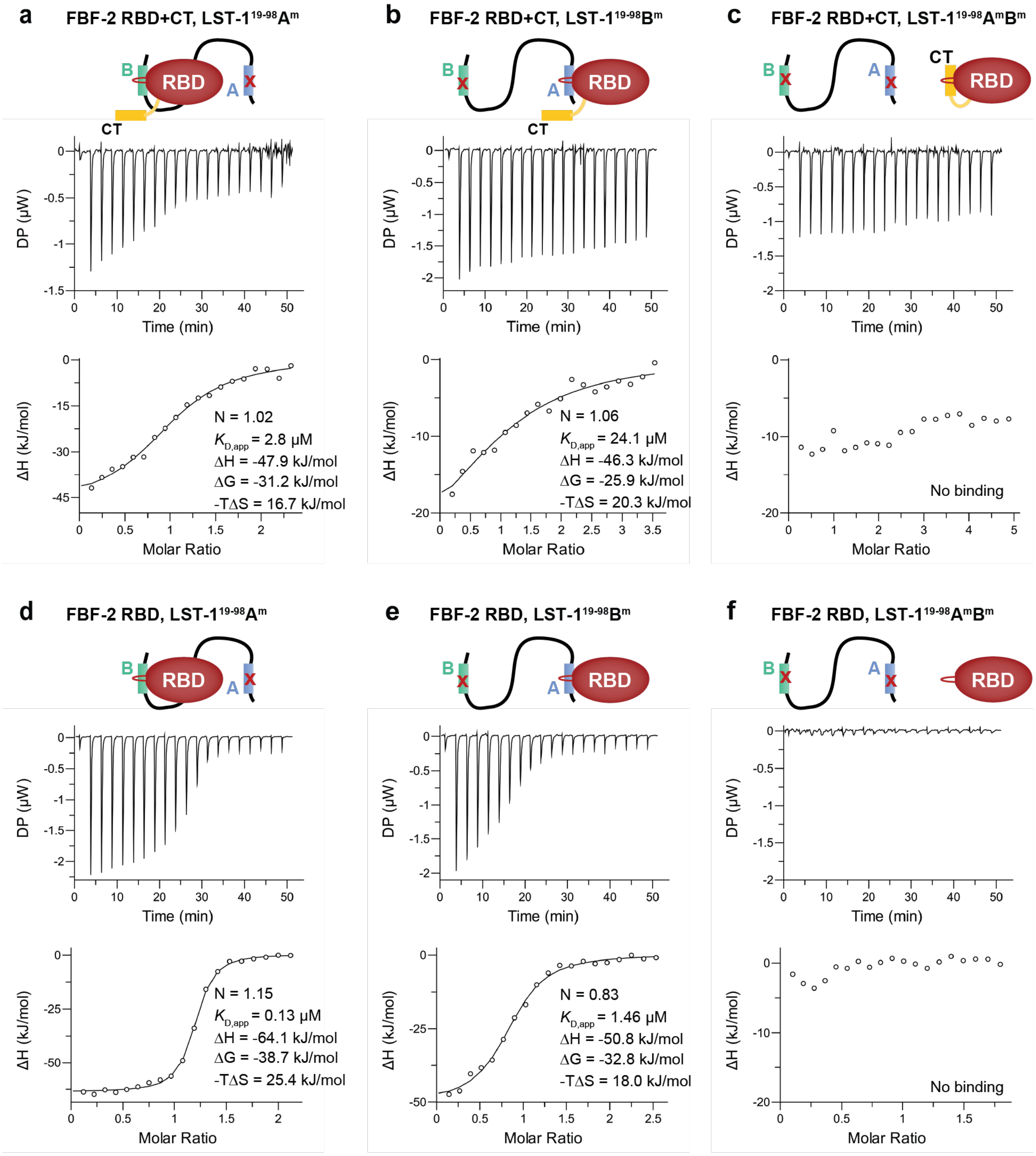

Supplementary Figure 1. LST-1 requires both PIMs for binding to two FBF-2 molecules. Representative ITC thermograms (above, differential power [DP] vs time) and corresponding titration curve-fitting graphs (below) for interaction of FBF-2 RBD+CT and **a** LST-1$^{19\text{-}98}$(A^{m}), **b** LST-1$^{19\text{-}98}$(B^{m}), and **c** LST-1$^{19\text{-}98}$ (A^{m}B^{m}). Representative ITC thermograms (above, DP vs time) and corresponding titration curve-fitting graphs (below) for interaction of FBF-2 RBD and **d** LST-

$1^{19\text{-}98}(A^m)$, **e** LST-$1^{19\text{-}98}(B^m)$, and **f** LST-$1^{19\text{-}98}(A^mB^m)$. Thermodynamic parameters from one replicate indicated in lower panels; thermodynamic parameters from two distinct technical replicates are presented in **Table 1**. Experimental components indicated in diagrams above graphs. Experiments that include FBF-2 RBD+CT (**a-c**) show residual heat that was not observed for those including FBF-2 RBD (**d-f**), suggesting this heat may be attributed to potential interactions with the FBF-2 CT.

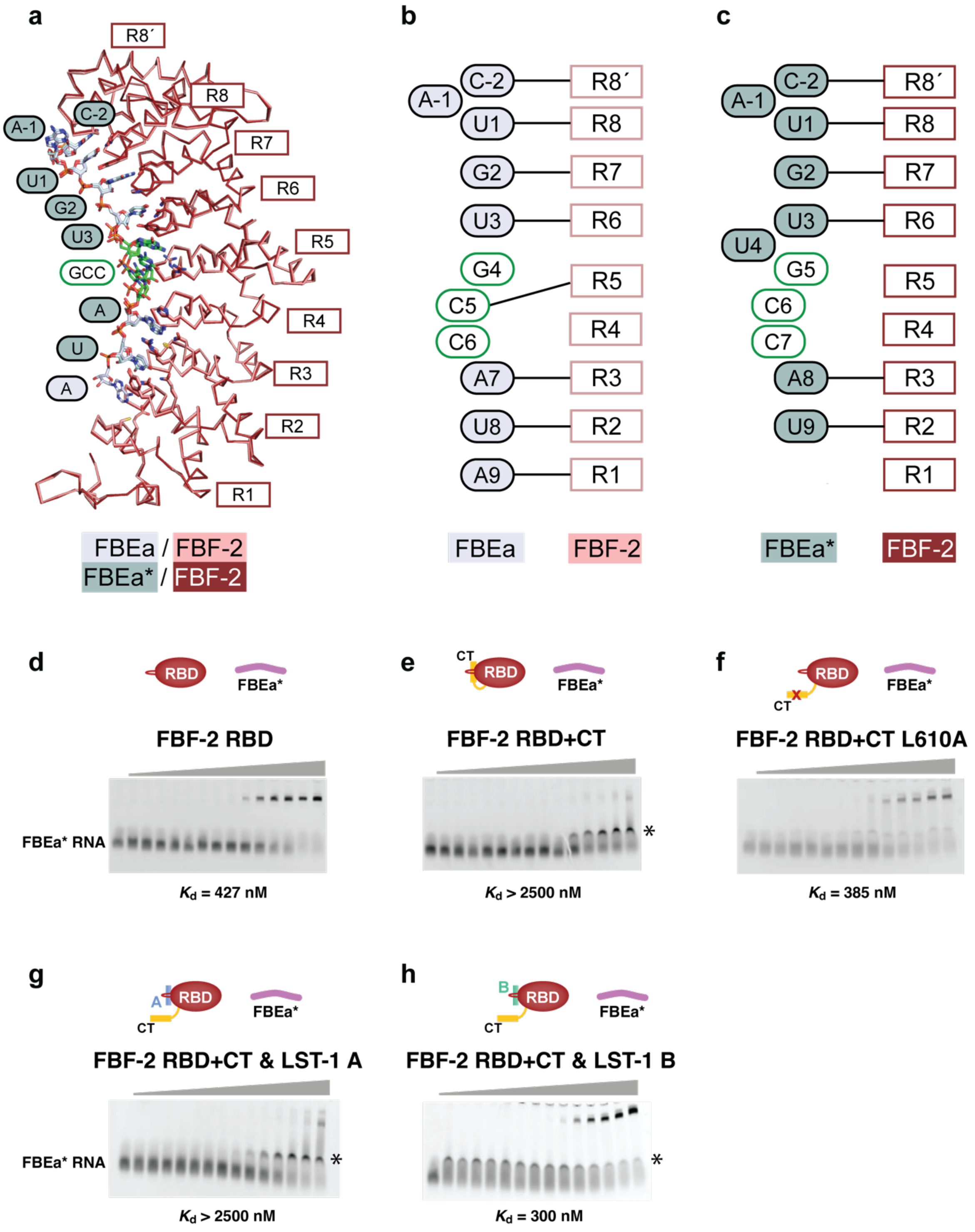

Supplementary Figure 2. FBF-2 binds to FBEa* RNA. **a** Superposition of crystal structures of FBF-2 RBD bound to *gld-1* FBEa RNA (PDB ID 3V74) or FBEa* RNA (PDB ID 8VIV). FBF-2 is

shown as CA traces with amino acid side chains that contact the RNA shown (pink, bound to FBEa; red, bound to FBEa*). The RMSD = 0.37 Å over 2,744 atoms in FBF-2. FBEa (pale blue) and FBEa* (pale cyan) are shown as stick models. **b, c** Schematic drawings of interactions between FBF-2 repeats and FBEa (**b**) and FBEa* (**c**) RNAs. A line between an RNA oval and an FBF-2 repeat rectangle represents at least one hydrogen bond or stacking interaction between amino acid side chains and the RNA base. Representative EMSA gels are shown for binding to FBEa* RNA (5´-AUCAUGUGCCAUAC-3´) by **d** FBF-2 RBD, **e** FBF-2 RBD+CT, **f** FBF-2 RBD+CT L610A, **g** FBF-2 RBD+CT with 150 μM LST-1$^{19-50}$ carrying PIM A, and **h** FBF-2 RBD+CT with 50 μM LST-1$^{67-98}$ carrying PIM B. Experimental components indicated in diagrams above gels. In panels **e** and **g**, we observed an intermediate band (*) that appears to be a non-specific interaction of FBF-2 RBD+CT, which was not observed for RBD. Similarly, LST-1$^{67-98}$ binds non-specifically to the RNA in panel **h**. We previously identified similar bands for non-specific binding to shorter RNAs[1]. We included these bands as part of the unbound RNA. Mean K_d values from at least three distinct technical replicates are reported. See also **Fig. 2f** and Source Data.

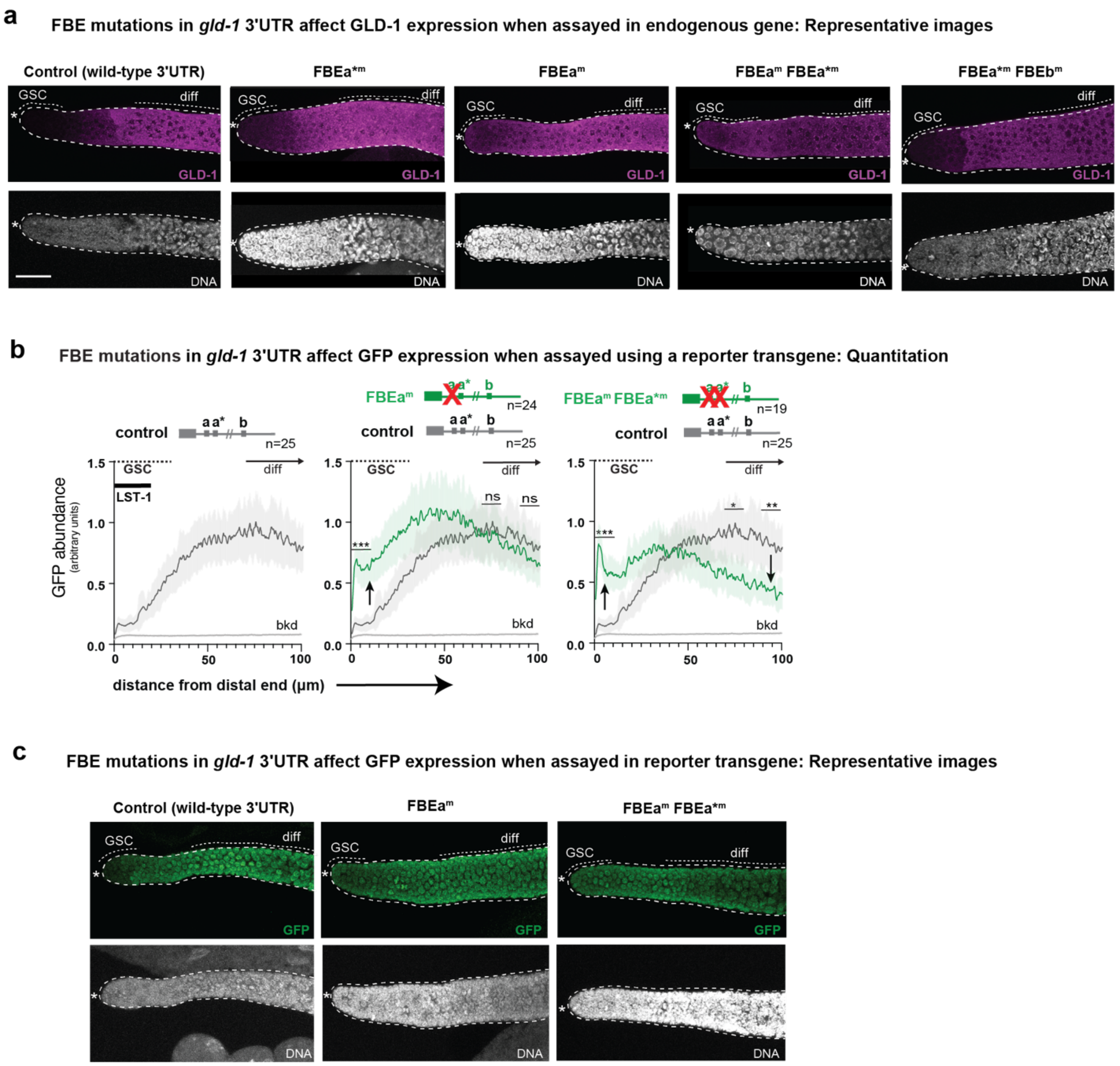

Supplementary Figure 3. Supplementary information for *gld-1* FBE mutants. **a** Representative z-projections of GLD-1 staining of FBE mutations generated in endogenous *gld-1*. Left to right: Thin dotted lines mark GSC pool and differentiation (diff); thick dotted line marks gonad boundary; asterisk marks distal end. Scale bar (bottom left), 20 μm for all panels in a and c. See Fig. 3d, e, h, i for quantitation. See Carrick et al.[2] for images of GLD-1 in FBEb^{m} and FBEa^{m}b^{m} mutants (graphs in Fig. 3g, j). **b** ImageJ quantitation of GFP abundance expressed from a *gld-1* 3´UTR reporter transgene as a function of position in the distal gonad. Gray lines show mean GFP pattern in wild-type; green lines show mean GFP pattern in mutant. Shading is the 95%

confidence interval. Wild-type and mutant data obtained from ‘n’ gonads processed and imaged together. Gonadal regions with GSCs (GSC) and differentiated (diff) germ cells are marked above; extent of LST-1 protein is marked with a thick black line in the control panel. P-values using a two-sided unpaired t-test assuming equal variance (no corrections for multiple comparisons) are given for pooled data in 0-10, 70-80 and 90-100 μm regions (black bars). P-values: *** $p < 0.001$, ** $p < 0.01$, * $p < 0.05$, ns (not significant) $p > 0.05$. See **Supplementary Table 2** for exact p-values. Left to right: Wild-type control (two replicates), FBEa^{m} (two replicates), and FBEa^{m} FBEa*^{m} double mutant (two replicates). Reporter constructs are shown above. Arrows indicate significant changes in GFP abundance relative to the wild-type control *gld-1* 3´UTR. Light gray line labeled “bkd” represents mean and 95% confidence interval for background staining in a wild-type animal not expressing the reporter construct (n=16, 1 replicate)[2]. **c** Representative z-projections of GFP reporter in extruded gonads with wild-type or mutated FBEs.

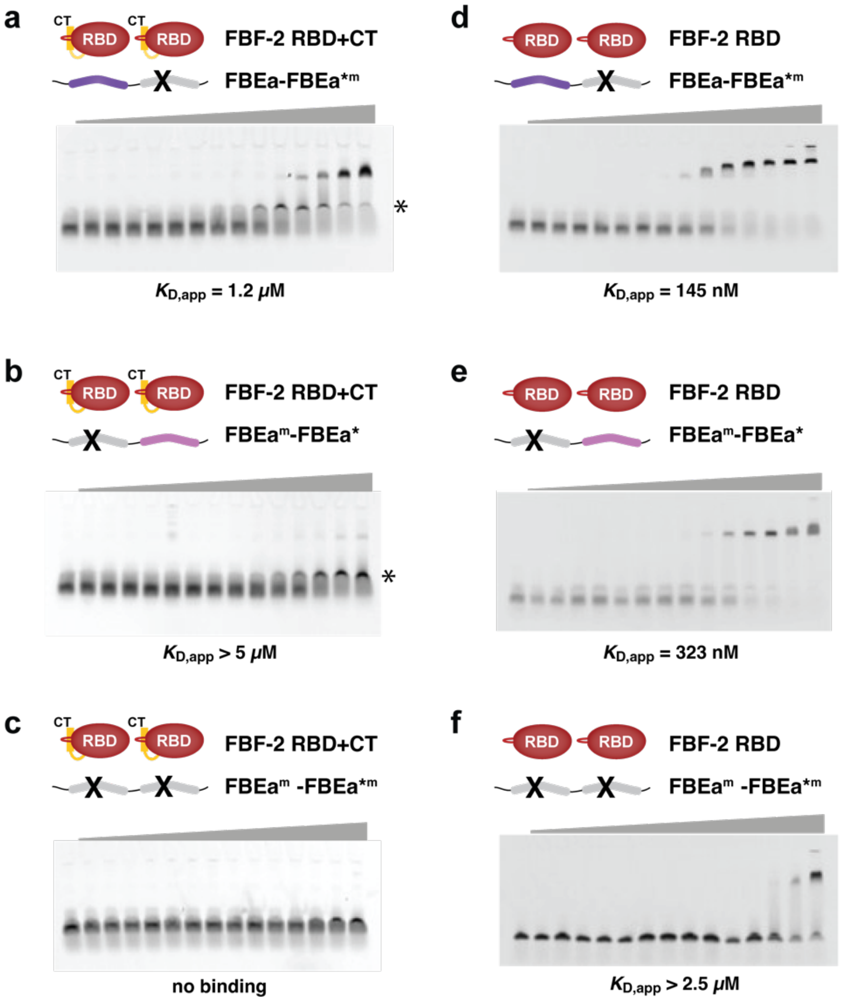

Supplementary Figure 4. Representative EMSA gels are shown for binding to FBEa-FBEa* RNA variants by **a-c** FBF-2 RBD+CT and **d-f** FBF-2 RBD. Experimental components indicated in diagrams above gels. LST-1 protein was not present. In panels a and b, we observed an intermediate band (*) that appears to be non-specific interaction of FBF-2 RBD+CT. We included these bands as part of the unbound RNA. Similar bands were detected previously with FBF-2 RBD+CT[1]. Mean K_d values from at least three distinct technical replicates are reported. See also **Table 2** and Source Data.

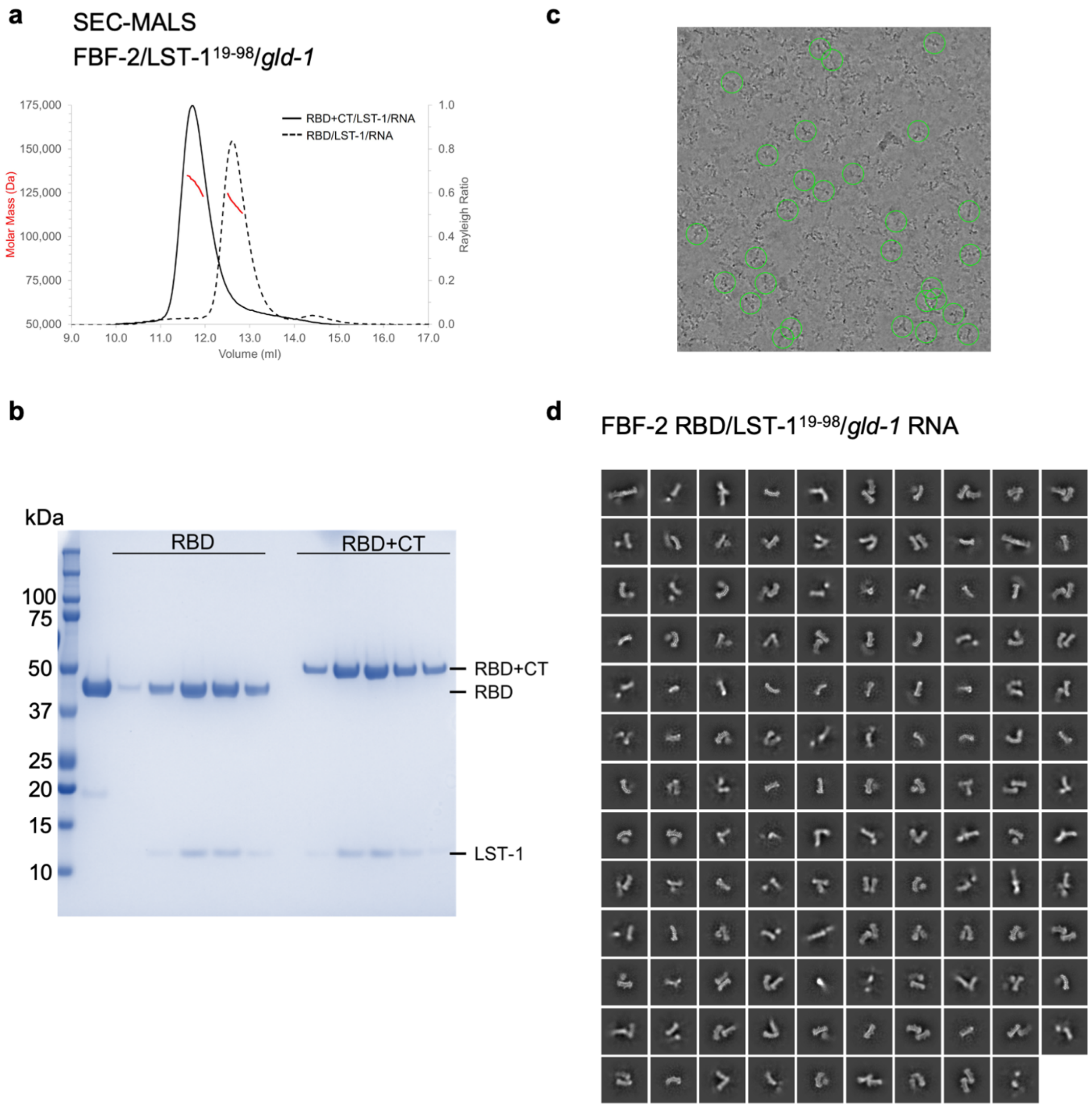

Supplementary Figure 5. Analyses of FBF-2/LST-1/ FBEa-FBEa* RNA complexes. **a** SEC-MALS analysis of FBF-2/LST-1$^{19\text{-}98}$/ FBEa-FBEa* RNA quaternary complexes. The peak for complexes with FBF-2 RBD+CT (solid black) had an apparent molecular mass of 130 kDa (red), which matches the calculated molecular weight of 125 kDa for a 2:1:1 complex of FBF-2 RBD+CT/LST-1$^{19\text{-}98}$/FBEa-FBEa*. The peak for complexes with FBF-2 RBD (dashed black line) had an apparent molecular mass of 119 kDa (red), which matches the calculated molecular weight of 119 kDa for a 2:1:1 complex of FBF-2/LST-1$^{19\text{-}98}$/FBEa-FBEa*. Molecular weights for individual components: FBF-2 RBD+CT, 53 kDa; FBF-2 RBD, 47 kDa; LST-1$^{19\text{-}98}$, 9.4 kDa, and

FBEa-FBEa* RNA, 8.6 kDa. **b** Coomassie-stained gel of SEC-MALS peak fractions. **c** Representative cryo-EM micrograph with locations of selected particles (green circles). **d** All 2D classes used for 3D reconstructions.

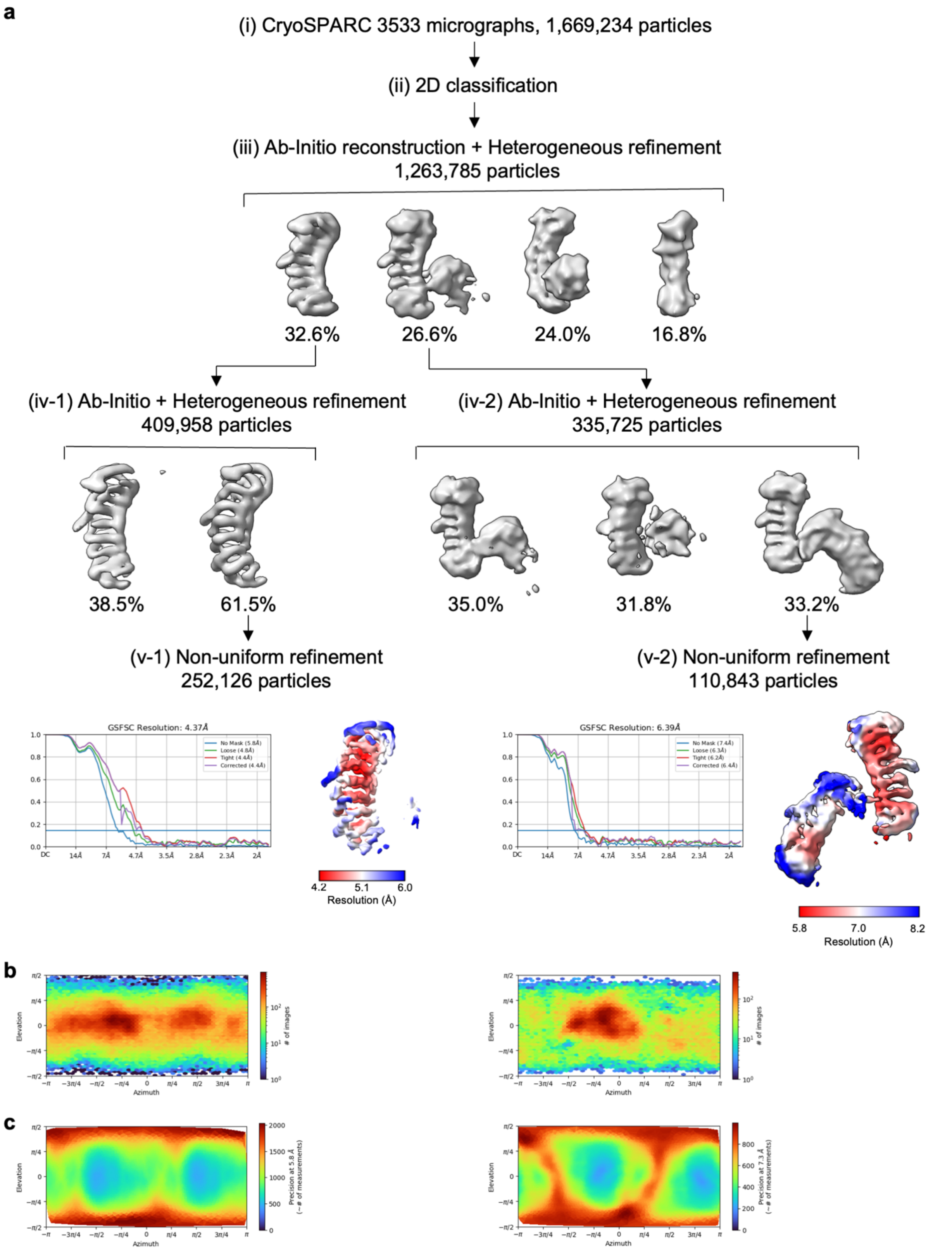
a
(i) CryoSPARC 3533 micrographs, 1,669,234 particles
(ii) 2D classification
(iii) Ab-Initio reconstruction + Heterogeneous refinement
1,263,785 particles
32.6%
26.6%
24.0%
16.8%
(iv-1) Ab-Initio + Heterogeneous refinement
409,958 particles
(iv-2) Ab-Initio + Heterogeneous refinement
335,725 particles
38.5%
61.5%
35.0%
31.8%
33.2%
(v-1) Non-uniform refinement
252,126 particles
(v-2) Non-uniform refinement
110,843 particles
GSFSC Resolution: 4.37Å
No Mask (5.8Å)
Loose (4.8Å)
Tight (4.4Å)
Corrected (4.4Å)
4.2
5.1
6.0
Resolution (Å)
GSFSC Resolution: 6.39Å
No Mask (7.4Å)
Loose (6.3Å)
Tight (6.2Å)
Corrected (6.4Å)
5.8
7.0
8.2
Resolution (Å)
b
Elevation
Azimuth
# of images
c
Elevation
Azimuth
Precision at 5.8 Å (~# of measurements)
Precision at 7.3 Å (~# of measurements)

Supplementary Figure 6. Cryo-EM reconstructions. **a** Overview of cryo-EM processing scheme of FBF-2/LST-1/ FBEa-FBEa* RNA complex. Details are described in the Methods. Fourier shell correlation (FSC) curves and maps colored based on local resolution for single FBF-2 (left) or 2 FBF-2 (right) reconstructions shown below. **b** Angular distribution of particles for single FBF-2 (left) or 2 FBF-2 (right) reconstructions. **c** Posterior precision directional distribution of particles for single FBF-2 (left) or 2 FBF-2 (right) reconstructions.

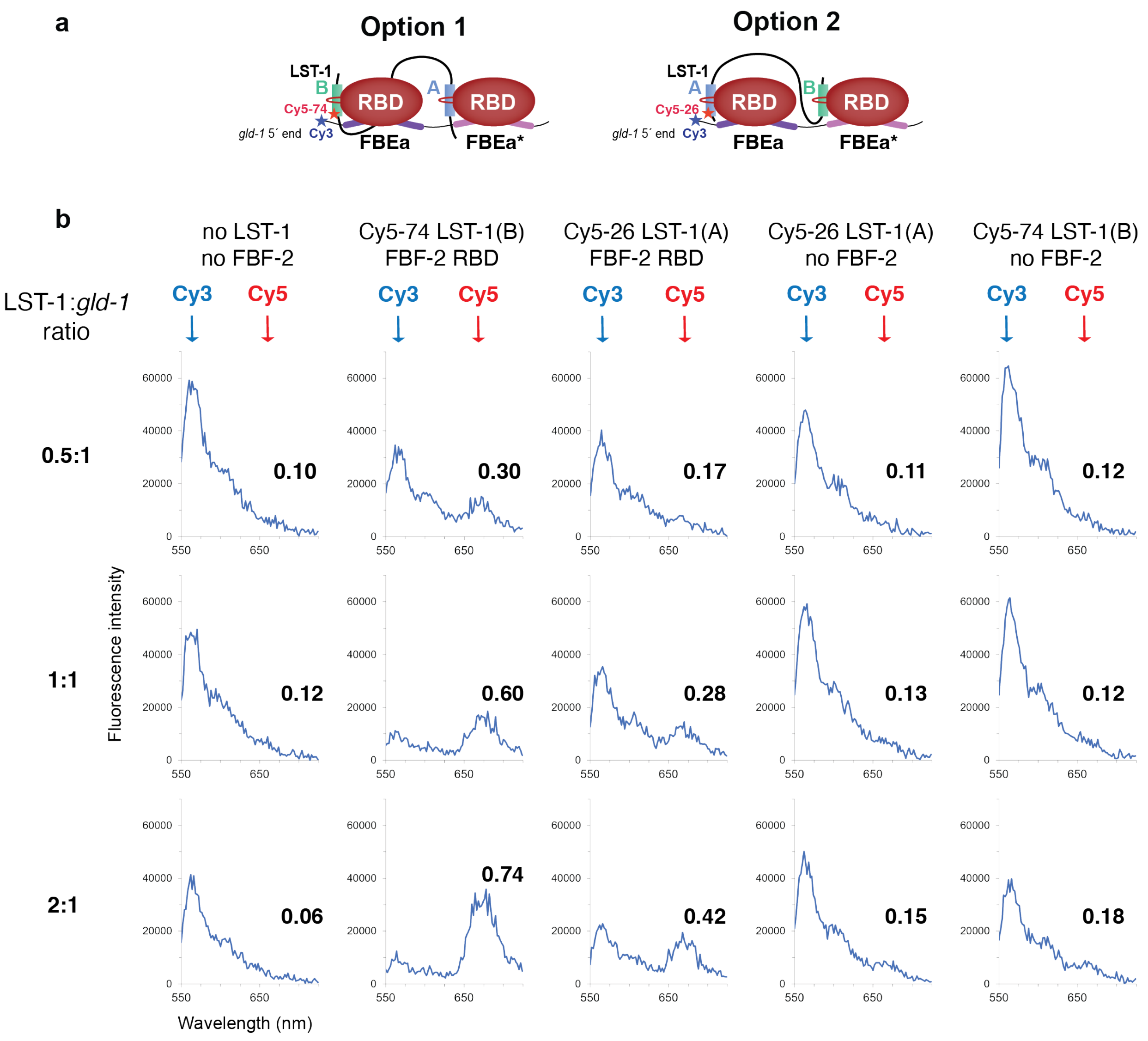

Supplementary Figure 7. FRET analysis of FBF-2/LST-1/ FBEa-FBEa* RNA complex. **a** Two possible orientations of LST-1 in the FBF-2/LST-1/ FBEa-FBEa* RNA complex. **b** Fluorescence spectra of excitation of Cy3-labeled RNA (blue arrow) and transfer to Cy5-labeled LST-1 (red arrow). In addition to complexes composed of Cy5-labeled LST-1, unlabeled FBF-2 RBD, and 5´-Cy3 labeled *gld-1* FBEa-FBEa* RNA, we also measured background levels of emission at 668 nm with samples of RNA only or RNA with LST-1 in the absence of FBF-2. FRET efficiencies were calculated as $I_{668}/(I_{668} + I_{564})$.

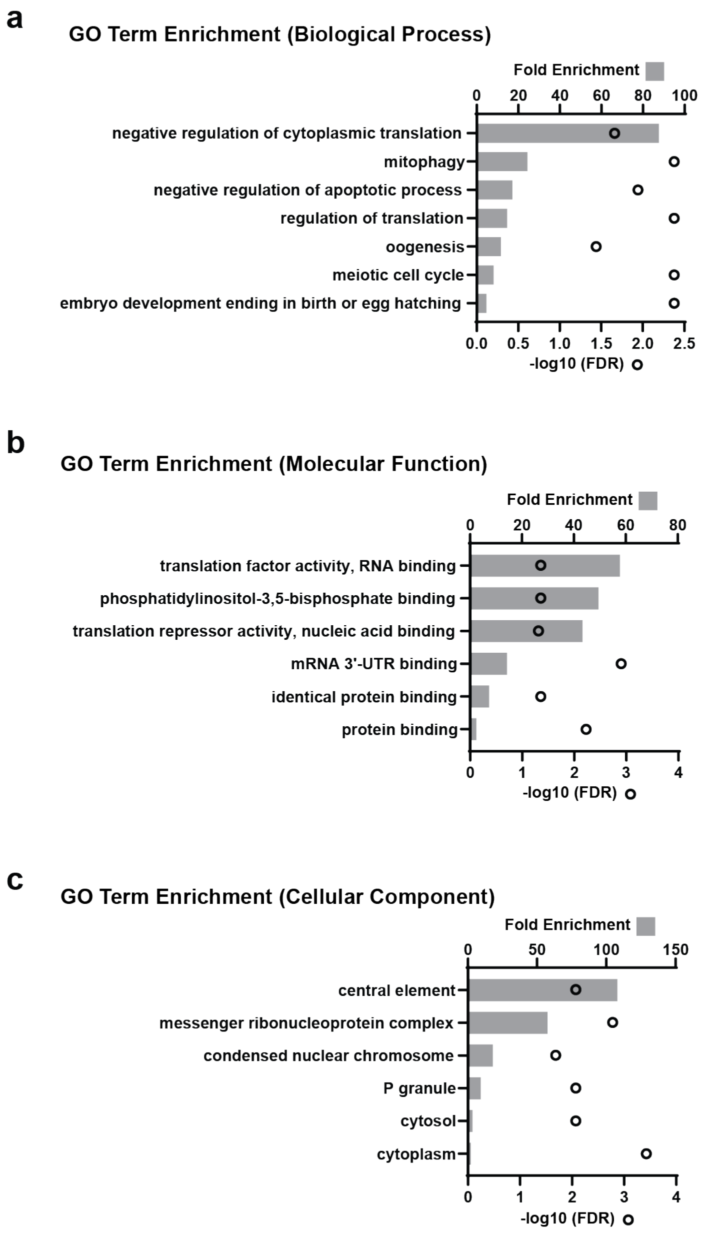

Supplementary Figure 8. GO term analysis of RNAs with adjacent FBEs. **a** Biological process GO term enrichment of genes that contain a peak with adjacent sites. Bars represent fold enrichment over background (top x-axis). Dots represent false discovery rate (FDR, bottom x-axis). Cutoffs for GO terms: fold enrichment ≥ 2 and FDR ≤ 0.05. **b** Molecular function GO term enrichment. Conventions and cutoffs as in **a**. **c** Cellular component GO term enrichment. Conventions and cutoffs as in **a**.

Supplementary References

1. Qiu C, Zhang Z, Wine RN, Campbell ZT, Zhang J, Hall TMT. Intra- and inter-molecular regulation by intrinsically-disordered regions governs PUF protein RNA binding. *Nat Commun* **14**, 7323 (2023).

2. Carrick BH*, et al.* PUF partner interactions at a conserved interface shape the RNA-binding landscape and cell fate in *Caenorhabditis elegans*. *Dev Cell* **59**, 661-675.e661-e667 (2024).
