# Supplementary material for: A higher order PUF complex is central to regulation of C. elegans germline stem cells
Source: Nat Commun. 2025 Jan 2;16:123. doi: 10.1038/s41467-024-55526-x (PMC11696143; doi:10.1038/s41467-024-55526-x)
Supplement: Supplementary file 3 — Description of Additional Supplementary Files [file 41467_2024_55526_MOESM3_ESM.pdf]

Description of Additional Supplementary Files

Supplementary Data 1: Closely spaced adjacent FBF-2 binding sites identified in analysis of eCLIP data.
