# Supplementary material for: A higher order PUF complex is central to regulation of C. elegans germline stem cells
Source: Nat Commun. 2025 Jan 2;16:123. doi: 10.1038/s41467-024-55526-x (PMC11696143; doi:10.1038/s41467-024-55526-x)
Supplement: Supplementary file 5 — Reporting Summary [file 41467_2024_55526_MOESM5_ESM.pdf]

nature portfolio

Corresponding author(s): Traci Hall

Last updated by author(s): Nov 20, 2024

Reporting Summary

Nature Portfolio wishes to improve the reproducibility of the work that we publish. This form provides structure for consistency and transparency in reporting. For further information on Nature Portfolio policies, see our Editorial Policies and the Editorial Policy Checklist.

Statistics

For all statistical analyses, confirm that the following items are present in the figure legend, table legend, main text, or Methods section.

n/a	Confirmed	
☐	☒	The exact sample size (n) for each experimental group/condition, given as a discrete number and unit of measurement
☐	☒	A statement on whether measurements were taken from distinct samples or whether the same sample was measured repeatedly
☐	☒	The statistical test(s) used AND whether they are one- or two-sided<br>*Only common tests should be described solely by name; describe more complex techniques in the Methods section.*
☒	☐	A description of all covariates tested
☒	☐	A description of any assumptions or corrections, such as tests of normality and adjustment for multiple comparisons
☐	☒	A full description of the statistical parameters including central tendency (e.g. means) or other basic estimates (e.g. regression coefficient) AND variation (e.g. standard deviation) or associated estimates of uncertainty (e.g. confidence intervals)
☐	☒	For null hypothesis testing, the test statistic (e.g. F, t, r) with confidence intervals, effect sizes, degrees of freedom and P value noted<br>*Give P values as exact values whenever suitable.*
☒	☐	For Bayesian analysis, information on the choice of priors and Markov chain Monte Carlo settings
☒	☐	For hierarchical and complex designs, identification of the appropriate level for tests and full reporting of outcomes
☒	☐	Estimates of effect sizes (e.g. Cohen's d, Pearson's r), indicating how they were calculated

Our web collection on statistics for biologists contains articles on many of the points above.

Software and code

Policy information about availability of computer code

Data collection	X-ray diffraction data were collected using SERGUI (SER-CAT beamline, APS).<br>ITC data were collected using the MicroCal PEAQ-ITC Automated Control Software (Version 1.40).<br>Images of germlines were collected using Leica Application Suite X v 3.5.7 image acquisition software.<br>Cryo-EM data were collected using Serial EM.<br>FRET data were collected using TECAN iControl (version 2.0).
Data analysis	X-ray diffraction data sets were scaled with HKL2000 (HKL Research).<br>Molecular replacement was performed with Phaser in Phenix version 1.20-4459.<br>X-ray crystallographic refinement was performed with Phenix version 1.20-4459.<br>Manual model building was performed with Coot (version 0.9.6 EL).<br>ITC data were analyzed using the MicroCal PEAQ-ITC Analysis Software (version 1.22).<br>Images of germlines were quantitated and prepared for figures using Fiji/ImageJ v 2.14.0.<br>Image quantitation was analyzed with Graphpad Prism v 9.4.1 - 10.2.2.<br>EMSA band intensities were quantified with ImageQuant 5.2 (Cytiva).<br>EMSA data were fit with GraphPad Prism (version 9.2.0).<br>SEC-MALS data were analyzed using ASTRA software (version 7.3.2.17).<br>Cryo-EM data were processed using CryoSparc (version 4).<br>Search for eCLIP adjacent sites was performed with Biopython 1.81.<br>Statistical analysis of eCLIP adjacent sites was calculated with GraphPad Prism 10.0.3.

For manuscripts utilizing custom algorithms or software that are central to the research but not yet described in published literature, software must be made available to editors and reviewers. We strongly encourage code deposition in a community repository (e.g. GitHub). See the Nature Portfolio guidelines for submitting code & software for further information.

Data

Policy information about availability of data

All manuscripts must include a data availability statement. This statement should provide the following information, where applicable:

- Accession codes, unique identifiers, or web links for publicly available datasets
- A description of any restrictions on data availability
- For clinical datasets or third party data, please ensure that the statement adheres to our policy

Data Availability
Lead contact
Further information and requests for resources and reagents should be directed to and will be fulfilled by the lead contacts, Traci Hall (hall4@niehs.nih.gov) and Judith Kimble (kimble@wisc.edu). Source data are provided with this paper.
Materials availability
Plasmids generated in this study will be deposited to Addgene.
Worm strains are available from the Caenorhabditis stock center.
Data and code availability
• Atomic coordinates and structure factors for the reported crystal structure have been deposited with the Protein Data Bank under accession number 8VIV and will be publicly available as of the date of publication.
• Cryo-EM maps have been deposited at the Electron Microscopy Data Bank under the accession numbers EMD45096 and EMD45097 and will be publicly available as of the date of publication.
• This paper analyzes existing, publicly available eCLIP data deposited at GEO under accession number GEO: GSE233561.
• This paper does not report original code.
• Any additional information required to reanalyze the data reported in this paper is available from the lead contact upon request.

Research involving human participants, their data, or biological material

Policy information about studies with human participants or human data. See also policy information about sex, gender (identity/presentation), and sexual orientation and race, ethnicity and racism.

Reporting on sex and gender	Not applicable, no human participants, data, or biological material were used.
Reporting on race, ethnicity, or other socially relevant groupings	Not applicable
Population characteristics	Not applicable
Recruitment	Not applicable
Ethics oversight	Not applicable

Note that full information on the approval of the study protocol must also be provided in the manuscript.

Field-specific reporting

Please select the one below that is the best fit for your research. If you are not sure, read the appropriate sections before making your selection.

☒ Life sciences ☐ Behavioural & social sciences ☐ Ecological, evolutionary & environmental sciences

For a reference copy of the document with all sections, see nature.com/documents/nr-reporting-summary-flat.pdf

Life sciences study design

All studies must disclose on these points even when the disclosure is negative.

Sample size	For in vitro assays: EMSAs were conducted at least in triplicate and values are reported as Kd ± SEM. This is a customary sample size, which provides the power to detect statistically significant differences, if present. ITC was conducted in duplicate due to the high amount of sample required for an individual replicate, and values for both experiments, which are similar, are reported.<br>For GLD-1 protein quantitation, we imaged 10-15 germlines per replicate. In some cases the number per replicate was lower for technical reasons.
Data exclusions	No data were excluded for in vitro assays or germline imaging. We did not image germlines with poor morphology.
Replication	Technical replicates for EMSAs and ITC produced similar results.<br>Two or three replicates were done for germline imaging using animals processed and imaged in separate experiments.
Randomization	For germline imaging, we randomly picked 20-30 animals for experiments from plates containing hundreds of animals.

Blinding	For in vitro assays, we did not blind the data processing from the data collection. Chen Qiu performed experiments and also processed the data.<br>For germline imaging, we did not blind quantitation or data analysis. Sarah Crittenden, Steph Dos Santos and Jennifer Woodworth did the experiments and data processing.

Reporting for specific materials, systems and methods

We require information from authors about some types of materials, experimental systems and methods used in many studies. Here, indicate whether each material, system or method listed is relevant to your study. If you are not sure if a list item applies to your research, read the appropriate section before selecting a response.

Materials & experimental systems

n/a	Involved in the study
☐	☒ Antibodies
☒	☐ Eukaryotic cell lines
☒	☐ Palaeontology and archaeology
☐	☒ Animals and other organisms
☒	☐ Clinical data
☒	☐ Dual use research of concern
☒	☐ Plants

Methods

n/a	Involved in the study
☒	☐ ChIP-seq
☒	☐ Flow cytometry
☒	☐ MRI-based neuroimaging

Antibodies

Antibodies used	Rabbit anti-GLD-1 from Dr. Elizabeth Goodwin lab Jan et al.,1997 (ref 69), 1:200<br>Mouse anti-GFP clone 3E6 ThermoFisher Scientific Catalog # A-11120 RRID:AB_221568, 1:200<br>Donkey-anti-mouse-Alexa 488 ThermoFisher Scientific Catalog #A21202 RRID:AB_141607, 1:1000<br>Donkey-anti-rabbit-Alexa 647 Jackson Immunoresearch Catalog # 711-605-152 RRID:AB_2492288, 1:1000
Validation	Low background levels of GLD-1 staining in gld-1(0) germlines (Suh et al., 2009 (ref 25), Fig 2)<br>Low background levels of GFP in control germlines not expressing GFP (Figure S3).

Animals and other research organisms

Policy information about studies involving animals; ARRIVE guidelines recommended for reporting animal research, and Sex and Gender in Research

Laboratory animals	Caenorhabditis elegans
Wild animals	The study did not involve wild animals
Reporting on sex	This study was done on hermaphrodite animals, which are easily identified by morphology and the presence of eggs in their uterus, because their regulation of GLD-1 is better understood than in males.
Field-collected samples	This study did not involve field-collected samples.
Ethics oversight	Ethical approval was not required for nematodes.

Note that full information on the approval of the study protocol must also be provided in the manuscript.

Plants

Seed stocks	Not applicable
Novel plant genotypes	Not applicable
Authentication	Not applicable
